# Dietary Sources, Sex, and rs5888 (*SCARB1*) as Modulators of Vitamin A’s Effect on Cardiometabolic Health

**DOI:** 10.3390/ijms241814152

**Published:** 2023-09-15

**Authors:** Sebastià Galmés, Andreu Palou, Francisca Serra

**Affiliations:** 1 Laboratory of Molecular Biology, Nutrition and Biotechnology (Group of Nutrigenomics, Biomarkers and Risk Evaluation−NuBE), University of the Balearic Islands (UIB), 07122 Palma, Spain; s.galmes@uib.cat (S.G.); francisca.serra@uib.es (F.S.); 2 Health Research Institute of the Balearic Islands (IdISBa), 07120 Palma, Spain; 3 CIBER of Physiopathology of Obesity and Nutrition (CIBEROBN), Instituto de Salud Carlos III, 28029 Madrid, Spain

**Keywords:** cardiometabolic health, vitamin A, sexual dimorphism, gene–diet interaction, PBMC

## Abstract

Although preclinical studies have attributed vitamin A (VA) cardiometabolic benefits, these effects are still controversial and not always supported in large human studies. Here, the outcomes associated with VA and its relationship with habitual dietary sources, sex, and genetic background have been studied. To do so, the data from an observational study (*n* = 455) (64% females, mean age of 36 years) showing that suboptimal VA intake (mainly from retinol rather than carotene) is associated with cardiometabolic risk (CMR) were considered. A higher odds ratio (OR) of suffering ≥ 2 simultaneous CMR factors was observed in men in the low consumption tercile of retinol (OR = 2.04; *p* = 0.019). In women, however, this relationship was not evident. Then, incubation of peripheral blood mononuclear cells (PBMCs) with VA-related compounds (ex vivo functional assay from 81 men and women) induced specific changes in the activity of genes involved in lipid homeostasis and inflammatory status, which were dependent on the type of compound tested and the sex of the person. In addition, the presence of the genetic variant rs5888 in *SCARB1* was identified as having a high influence on VA-related metabolic response. The new evidence derived from this study could be relevant for personalized nutritional advice concerning VA and CMR.

## 1. Introduction

Cardiovascular diseases are the leading cause of mortality, accounting for 31% of total deaths worldwide [1]. The sustained increase over decades has been linked to the westernization of diet [2], characterized by processed and high-energy-dense foods with a shortage of fruits, vegetables, and other nutrient-rich foods [3]. In this scenario, adequate intake of bioactive compounds can be relevant in cardiometabolic risk (CMR) prevention strategies. Specifically, carotenoids and vitamin A (VA) are essential micronutrients with proven cardiometabolic benefits shown in preclinical studies, including, among many others, anti-obesity, anti-inflammatory, and antioxidant potential [4]. Most of the mechanisms of action underlying the beneficial effects of VA-related compounds have been suggested to involve a physical interaction with plasmatic and/or nuclear target molecules. Specifically, VA and its specific precursors can interact with nuclear receptors and modulate the expression of crucial genes involved in the cellular redox and inflammatory status, for example by activating the nuclear erythroid 2-related factor 2 (NRF2) and/or inhibiting the nuclear factor κB (NFΚB) pathways (reviewed in [4]). β-Carotene (BC) is the most abundant carotenoid in the human diet. Once ingested, some carotenoids (including BC) can be enzymatically converted to retinol (R-OH) and then irreversibly oxidized to retinoic acid (RA) [4]. In addition, preformed R-OH can be obtained from animal products, mainly as retinyl-esters, which are more efficiently convertible to RA than BC [5]. Although R-OH and carotenes can directly modulate cell metabolism [4], RA is considered the VA-active form. The vast majority of in vitro and in vivo studies suggest a RA role in modulating mammal lipid homeostasis and energy metabolism [6,7]. Nevertheless, population studies may show some disparities between VA consumption (and related compounds) and health outcomes [8]. Moreover, sexual dimorphism in VA metabolism is glimpsed in animal models [9], although this evidence has not been corroborated in human studies. Furthermore, genetic variants involved in carotenoid and VA cellular handling have been associated with the variability found in VA circulating levels in humans, which might compromise its bioavailability and metabolic responsiveness [10]. Therefore, potential interactions between these factors need to be addressed to optimize healthier dietary patterns that target individual needs with higher precision.

Accordingly, in this study, we aimed to assess the differential impact of VA and precursors on cardiometabolic health in the general population, considering their dietary sources together with the genetic characteristics and sex of the subjects. For that purpose, data from two complementary studies were analyzed. A cross-sectional study was used to assess the association between VA intake (and dietary sources) and the presence of the following CMR factors: high percentage of body fat (BF%), visceral obesity (VO), hypertension (HT), dyslipidemia (DL), and type 2 diabetes (T2D), and their simultaneous suffering. Then, RA, R-OH, and BC ex vivo impacts on the expression of lipid homeostasis, inflammatory response, and oxidative status key genes were explored in peripheral blood mononuclear cells (PBMCs) from a smaller cohort, age- and sex-paired. The results will provide novel insights to better harness the health benefits of VA and be useful for addressing more targeted personalized nutrition strategies to prevent CMR and improve human health. 

## 2. Results

### 2.1. High Intake Level of Vitamin A Is Associated with Benefits for Cardiovascular Health, Particularly in Men

**Figure 1:**
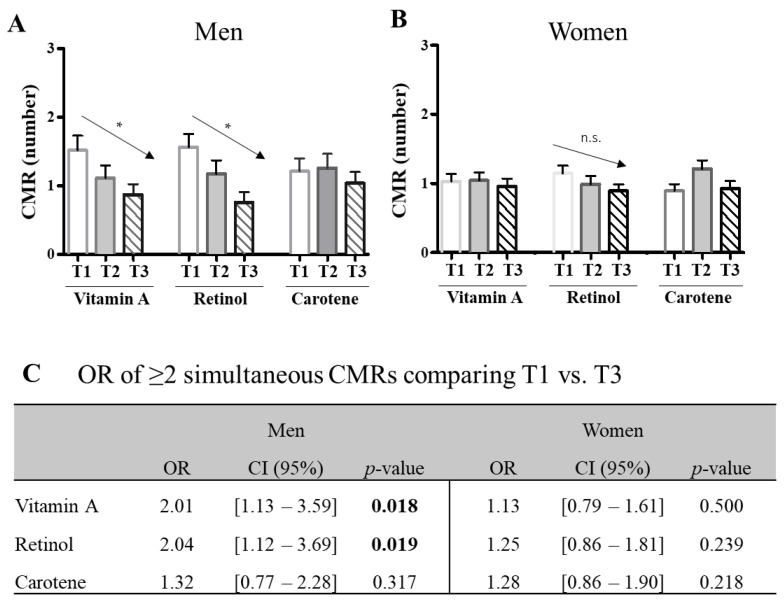
Presence of cardiometabolic risk (CMR) factors in relation to terciles (T1, T2, and T3) of intake of vitamin A, retinol, and carotene in men (**A**) and women (**B**). The association between the number of CMR factors (mean ± standard error) and intakes was assessed by linear regression, adjusting for age, energy consumed, and level of physical activity. *p*-values < 0.05 are indicated by (*), and n.s. means no statistical significance. The odds ratio (OR) and 95% confidence interval (CI) of suffering two or more simultaneous CMR factors between T1 and T3 (**C**) were assessed by binary logistic regressions (adjusted for the same covariates) and using the T3 of each nutrient as a reference (OR = 1). Significant *p*-values (<0.05) are indicated in bold.

The Ob-IB cohort was a sample (*n* = 455) based on the general population of Mallorca (mean age 36), involved a higher (63.7%) participation of women, and almost half of the population showed a high body fat percentage (BF%) (47.0%), with similar percentages in men and women. The prevalence of visceral obesity (VO) was also high (34.1%) but aligned with the Spanish population data [11]. Hypertension (HT) prevalence (9.4%) was higher in men (14.2%) than in women (6.7%), as was the incidence of type 2 diabetes (T2D), which was 5.4% in the general population, 8.1% in men, and 3.9% in women. In contrast, the frequency of dyslipidemia (DL) (11.2%) was similar between sexes (11.7% in men; 10.9% in women). On average, 30.6% of the subjects were suffering from ≥2 CMR factors simultaneously (33.5% of men; 28.9% of women) (Table 1). The reported diet was high in lipids (37.7% energy) and low in carbohydrates (42.3% energy). Average VA consumption showed high variability and was above dietary recommendations in men (148%) and women (126%) [12]. Men’s retinol intake nearly doubled (686 ± 3637 Eq/day) compared to women’s (353 ± 811 Eq/day); no differences were found in carotenes (1920 ± 1665 µg/day in men, 2254 ± 2112 µg/day in women) (Table 1). Then, the population was distributed into terciles of consumption, and linear regression tests showed that terciles of VA and retinol intake were inversely associated with the presence of CMR factors in men but not in women (β = 0.141; *p* = 0.023, for VA; β = 0.138; *p* = 0.027, for retinol). No relationship was observed for carotene intake level. The odds ratio (OR) of suffering ≥2 simultaneous CMR factors confirmed a higher risk in men in the lowest tercile (T1 vs. T3) of VA (OR = 2.01; *p* = 0.018) or retinol (OR = 2.04; *p* = 0.019). In contrast, low intake of VA, retinol, or carotene was not linearly or significantly associated with CMR in women (Figure 1). 

### 2.2. Vitamin A and Precursors Regulate Ex-Vivo PBMC Gene Expression in a Sex-Dependent Manner

Next, eighty-one people were recalled and included in the ex vivo study (OptiDiet-15 cohort). Age balance (≈36 for both sexes), equal participation (41 men, 40 women), and a similar CMR number (1.22 vs. 1.15) were achieved between the sexes. Isolated PBMCs were incubated with RA and precursors to assess the metabolic flexibility of the subjects on cellular lipid management and inflammation-oxidation processes. All the treatments modulated gene expression in PBMCs, with the effect being more intense in women than in men, and cells from men were less responsive to BC in comparison with women. 

**Figure 2:**
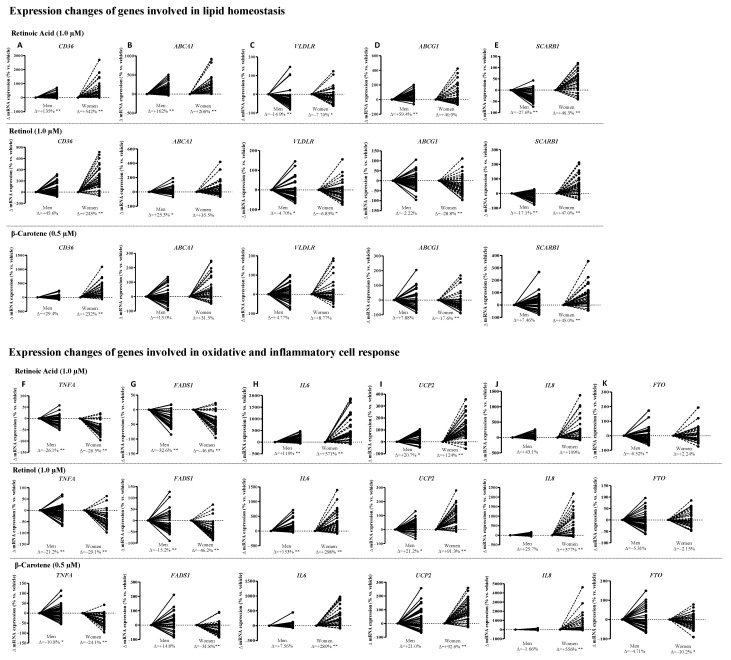
Effect of retinoic acid, retinol, and β-carotene treatments on mRNA expression of genes involved in lipid homeostasis (**A**–**E**) and in oxidative and inflammatory cell response (**F**–**K**) in peripheral blood mononuclear cells (PBMCs) from men (*n* = 41) and women (*n* = 40), volunteers of the OptiDiet-15 study. The data correspond to mRNA levels after 48 h of ex vivo incubation of PBMCs. The genes studied are the following: (**A**) cluster of differentiation 36 (*CD36*), (**B**) ATP-binding cassette transporter subfamily A1 (*ABCA1*), (**C**) very-low-density lipoprotein receptor (*VLDLR*), (**D**) ATP-binding cassette transporter subfamily G1 (*ABCG1*), (**E**) scavenger receptor class B member 1 (*SCARB1*), (**F**) tumor necrosis factor alpha (*TNFA*), (**G**) Δ-5 fatty acid desaturase 1 (*FADS1*), (**H**) interleukin 6 (*IL6*), (**I**) uncoupling protein 2 (*UCP2*), (**J**) interleukin 8 (*IL8*), and (**K**) fat mass and obesity-associated gene (*FTO*). The % change (∆) was obtained by the difference between the mean of gene expression in treated cells and the mean of gene expression under baseline conditions in each experimental group. A statistical assessment of the effect of the treatments on each sex was estimated using a paired Student *t*-test; *p* < 0.05 is indicated by (*), and *p* < 0.01 is indicated by (**).

Concerning the expression of lipid handling-related genes (Figure 2A–E), RA promoted the highest induction on *CD36* (543% in women and 135% in men) with respect to basal levels. A similar impact was observed with R-OH (243%) and BC (232%) only in women. Lower inductions were observed (range 29–46%) in men with the VA precursors. Expression of *ABCA1* was induced the most by RA (162% in men and 206% in women) and by R-OH in men (25.3%). In contrast, RA and R-OH decreased *VLDLR* expression in both sexes (range −5% to −15%). Sex-specific induction was observed in *ABCG1* mRNA in men by RA (59.4%) and in *SCARB1* with the three compounds in women (≈46%). Sex-specific inhibition by R-OH and BC was shown in *ABCG1* only in women (range −18 to −29%) and by RA and R-OH in *SCARB1* only in men (range −17 to −28%).

Regarding the expression of genes related to inflammation (Figure 2F–K), RA and R-OH increased *IL6* in men (119% and 153%, respectively) and were 5–3 times higher in women (571% for RA and 298% for R-OH). BC induced *IL6* expression only in women (289%). The three treatments induced *UCP2* expression to a similar extent in women (range: 91–124%) and to a lesser extent in men (≈20% for the three compounds tested). The treatments decreased *TNFA* expression to a similar extent in both sexes (range: −10.8% to −29.1%). *FADS1* expression was down-regulated under RA (−32.6% in men and −46.6% in women) and R-OH (−15.2% in men and −46.2% in women), but only in women under BC treatment (−31.8%). Sex-specific induction was observed for *IL8* expression in women’s PBMCs treated with R-OH (377%) and BC (556%) and tended to increase with RA (199%). However, none of the treatments caused significant changes in men (range: −1% to 43%). Finally, *FTO* expression decreased slightly in PBMCs from men with RA (−6.52%) and in women with BC (−10.2%).

### 2.3. Relevant Genetic Variants Can Modulate the Response to Carotene Intake/BC Exposure and Cardiometabolic Health

Haplotype analyses of five genetic variants related to cellular management of lipids (*SCARB1* and *CD36*) and inflammation–oxidative metabolism (*FTO*, *UCP2*, and *TNFA*) were studied in their relationship with carotene intake and cardiometabolic health. The top 10 most common haplotypes found in the Ob-IB cohort (Table 2) show that cardiometabolic health could be influenced by genetic variants interacting with the dietary intake of carotenes in a sex-dependent manner. The haplotypes with the highest CMR (vs. the most common, ref) differed in *SCARB1* and *FTO* alleles in men (17.8% of men) and in *SCARB1* and *CD36* in women (33.4% of women). Therefore, specific genotypes (mainly TACTG in men and TACAG in women) could set up certain metabolic disadvantages in carotenoid management, promoting higher cardiometabolic risk in low-carotene consumers. Interestingly, a higher intake (T3) of carotenoids would reverse the adverse effects on CMR and promote better health in those genotype carriers, at least in the general population. 

**Figure 3:**
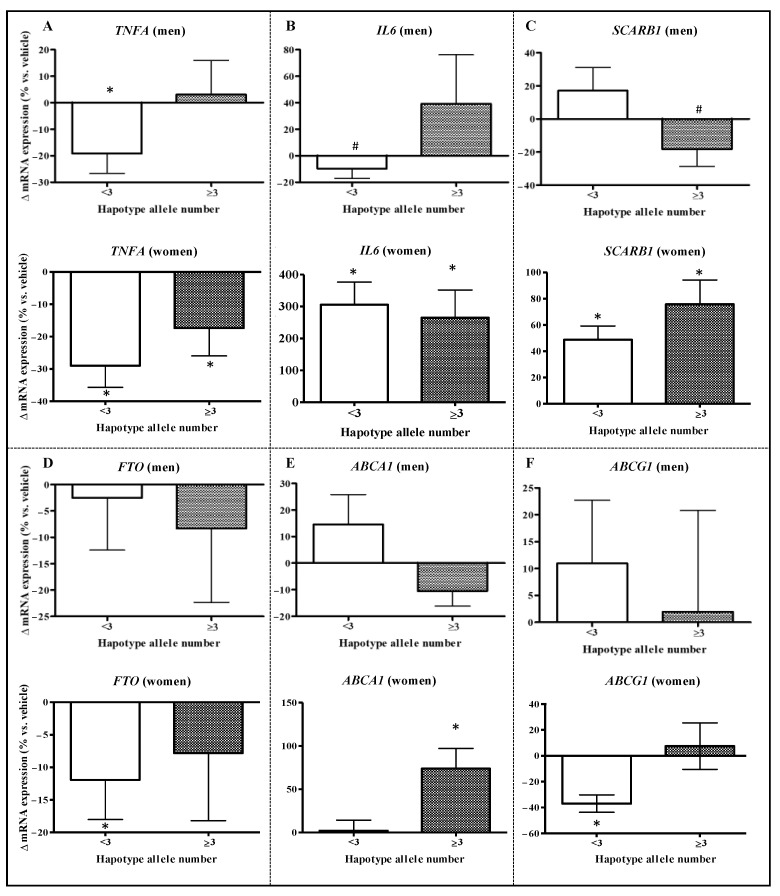
Changes in gene expression by β-carotene in comparison with control cells (treated with DMSO) by gender and haplotype group in peripheral blood mononuclear cells (PBMCs). Expression data are from ex vivo incubation of PBMCs during 48 h from women (*n* = 40) and men (*n* = 41) volunteers of the OptiDiet-15 study (total *n* = 81), grouped by the number of alleles associated with cardiometabolic health in the Ob-IB cohort. In men, the number of T alleles of both rs5888 (*SCARB1*) and rs9939609 (*FTO*) was added to determine the haplotype groups: low (<3 alleles, *n* = 26) and high (≥3, *n* = 15). In women, the number of alleles T in rs5888 (*SCARB1*) and A in rs1761667 (*CD36*) was added to determine the haplotype groups: low (<3 alleles, *n* = 23) and high (≥3, *n* = 17). Bars represent the mean of the change induced by β-carotene (Δ, increases if it is positive or decreases if it is negative) calculated in comparison with the respective control cells, and the whiskers represent the standard error of the mean (SE). Statistical differences in expression vs. control cells were performed by Student’s paired test: *p* < 0.05 is indicated by (*) and *p*-values between 0.05 and 0.06 are indicated by (^#^). Genes analyzed: (**A**) *TNFA* (tumor necrosis factor alpha), (**B**) *IL6* (interleukin 6), (**C**) *SCARB1* (scavenger receptor class B member 1); (**D**) *FTO* (fat mass and obesity-associated gene), (**E**) *ABCA1* (ATP-binding cassette A1), and (**F**) *ABCG1* (ATP-binding cassette G1).

To discern the influence of genetics, we reanalyzed gene expression in PBMCs stratified by genotype (low/high; cut-off: three alleles associated with potential better/adverse cardiometabolic response) and focused on the response to BC (Figure 3). Results confirmed the hypothesis that the presence of genetic variants plays a role in defining the response to BC, at least ex vivo, by modulating genes involved in lipid homeostasis and inflammatory processes in a sex-dependent manner.

In women, the influence of genetics in response to BC was observed in *FTO*, *ABCG1*, and *ABCA1* mRNA levels. However, in men, it was relevant for *TNFA*, *SCARB1*, and *IL6* expression. *FTO* and *ABCG1* decreased in women with low genetic load (−12.0% and −37.0%) but not in high carriers, which did not differ from basal expression levels (Figure 3D). In contrast, *ABCA1* expression was significantly increased (+73.8%) in women with ≥3 alleles (Figure 3E). In men, the expression of the pro-inflammatory cytokines *TNFA* and *IL6* showed a decrease associated with the BC treatment in low carriers (−19.2%, Figure 3A and −9.61%, Figure 3B) in comparison with high carriers. In contrast, *SCARB1* expression was unresponsive to BC in low carriers and promoted a slight decrease (−18.3%) in the PBMCs of high carriers (Figure 3C).

## 3. Discussion

Here we highlight the relevance of sex, the VA source (retinol or carotene), and the presence of genetic variants as elements that contribute to differential metabolic responses among subjects and have an impact on lipid metabolism and cardiometabolic health. Two complementary approaches contributed to this understanding: an ex vivo system based on the analysis of PBMC responses to VA and precursors and a cross-sectional study characterizing nutritional, genetic, and cardiometabolic traits in the general population. 

The ex vivo approach, based on the analysis of gene expression in PBMC, was carried out in an age-sex-matched cohort, an adult Caucasian-based population. The effects of VA-related compounds on metabolism and their relationship with cardiometabolic health and the genetics of the volunteers were tested. This methodological approach constitutes a very useful tool to evaluate inter-individual responsiveness to bioactives. Isolated PBMCs allow studying the metabolic flexibility of subjects at the cellular level [13] and the effectiveness of specific nutritional or therapeutic treatments [14]. Overall, the action of VA-related compounds modulated gene expression, aiming to promote better cellular flow of lipids and prevent their accumulation. Furthermore, the PBMC system was sensitive enough to reveal sex- and genetic-specific adaptations of gene transcription. 

VA-related compounds caused a remarkable induction in *CD36* expression in women and, to a lesser extent, in men. This gene is involved in the uptake of LDL and oxidized LDL [15], as well as of carotenes and tocopherols [16]. *CD36* deficiency is associated with atherosclerotic disease and foam cell production [17]. The induction of *CD36* works together with *VLDLR* downregulation (essentially by RA), which is also involved in VLDL internalization [18]. *VLDLR* expression is induced in atherosclerotic lesions [19] and cellular lipid over-accumulation [18]. *ABCA1* and *ABCG1* also contribute to preventing lipid over-accumulation and foam cell transformation [20]. Moreover, cholesterol release from macrophages (protecting from atherosclerotic plaque formation) promoted by RA treatment is mediated by the induction of *Abca1* and *Abcg1* expression in *ApoE*-deficient mice [21]. The link between *SCARB1* activity and cardiovascular benefits is unclear [22]. Its induction (as seen in women) could lead to improved handling of carotenoids and lipophilic vitamins [23], but the current controversy could be related to the opposite sex effect revealed by our data, together with the influence of the genetic background. Therefore, gene responses to VA precursors in PBMC could be understood as a beneficial effect that would agree with a protective effect against lipid over-accumulation, a more fluid transit of lipids, and a greater cellular capacity to uptake carotenes and other lipophilic essential substances [23].

TNF-α and IL6 are pro-inflammatory cytokines released to the media by PBMC in a pro-inflammatory environment [24]. VA precursors inhibited the expression of T*NFA* in both sexes. Previous studies have reported that RA reduces *TNFA* expression and protein production in PBMC [25], and our study successfully replicated these findings. Moreover, we found that R-OH and BC also down-regulate *TNFA* expression. In contrast, *IL6* was highly induced by the treatments in both sexes, apart from PBMC treated with BC in men, which were not responsive at all. This induction aligns with a previous study showing that RA prolongs the increased expression of *IL6* in macrophages following a pro-inflammatory stimulus, suggesting a better physiological response against infection agents [26]. Except for BC in men, the tested compounds caused significant changes in *FADS1* (a decrease) and *UCP2* (an increase). Considering *FADS1* is induced in inflammatory environments and plays a role in resolving inflammation [27], and *UCP2* activation may lead to cellular ROS buffering [28], these effects would reflect an improved redox cellular status. Despite the fact that *IL8* and *FTO* expressions were affected in a sex-compound-specific manner, an increase in *IL8* and a decrease in *FTO* would also be associated with a beneficial cellular state. In fact, higher *IL8* expression is reported in PBMCs from healthy subjects, in contrast with the lower levels found among metabolic syndrome patients [29], while elevated *FTO* expression has been associated with decreased hepatic lipolysis by modulating the methylation of genes involved in lipid metabolism [30]. Therefore, the activities observed by RA, R-OH in both sexes, and BC in women could lead towards a better inflammatory/redox status. Furthermore, they suggest a male-specific lower anti-inflammatory potential associated with BC that merits further characterization in other cell types.

Analysis of the main cohort showed that a higher intake of vitamin A (mostly from retinol rather than from carotene) is associated with a lower CMR. To identify individual factors that may affect the benefits of these compounds, genetic information was examined. Haplotype analysis identified specific combinations of alleles that were associated with a more negative impact on cardiometabolic health in individuals with low carotene intake (T1), particularly SNP rs5888. The presence of the T allele in this haplotype, which is shared by both genders, showed the greatest difference in CMR compared to the respective references. Interestingly, this allele has been associated with men’s lower risk of cardiovascular disease in a meta-analysis [31] but also with women’s increased risk of premature coronary artery diseases [32]. Our results add another layer of complexity, as *SCARB1* expression in response to VA showed sexual dimorphism, promoted induction in women, and caused repression in men, and the T allele was found to be associated with the worst cardiometabolic outcome. 

Re-analysis of the BC response based on haplotype characteristics has revealed sex-specific differences that become magnified in individuals with specific genotypes and would involve different metabolic flexibility from VA precursors. For example, under this new perspective, the anti-inflammatory response attributable to the decrease in *TNFA* under BC treatment was only established in PBMC from men with <3 alleles. The effect of BC in genotype-specific men may be related to a higher capacity for anti-inflammatory effects, while in women these effects remain controversial and would be more focused on lipid metabolism improvements. These distinct effects, depending on the sex, may be attributable to the hormonal pattern of each sex, so this condition plus the genotype background must be considered for the personalization of nutrition.

There are some limitations in the present study that should be noted to properly interpret the results. First, because of the cross-sectional design of the Ob-IB study, a selection bias may have been introduced, and the potential effect of other confounders (such as socio-economic factors or other genetic variants) was not considered. Second, the sample size of the Ob-IB cohort is relatively small for an observational study. Although the results are supported by the subsequent ex vivo study OptiDiet15, the notable sample size analyzed in that case may counteract the first point. Third, multiple testing restrictive corrections (FDR) were not applied to the statistical models performed because of the previously established hypothesis driven by the variables to be analyzed. Hereby, these results are of relevance concerning the cardiometabolic health of the general population and deserve further studies to replicate the findings in other cohorts.

In conclusion, the results obtained in this study bear significant potential for opening new avenues of inquiry. On the one hand, these findings could encompass more comprehensive studies in fundamental research, delving deeper into the underlying mechanisms of the analyzed genetic variants. Additionally, these results should guide the design of clinical studies to assess the impact of vitamin A and its precursors. Furthermore, this body of knowledge offers the prospect of early identification of individuals who may be at risk of developing cardiometabolic disorders in the event of suboptimal vitamin A nutritional status. This newfound understanding can be leveraged to tailor dietary recommendations more effectively, thereby promoting and safeguarding their overall health.

## 4. Materials and Methods

### 4.1. Study Design

The results included in this report are based on data from two complementary approaches, the Ob-IB and OptiDiet-15 studies, which were developed between September 2013 and May 2019. The protocols of both studies have been evaluated and approved by the ethics committee (Comitè d’Ètica de la Investigació de les Illes Balears, CEI-IB), and all procedures were in accordance with Helsinki Declaration principles. The Ob-IB study (IB 2009/13 PI) is a cross-sectional observational study aiming to characterize the general population of Mallorca (Balearic Islands, Spain) in nutritional, anthropometric, and genetic terms. The volunteers for these studies were recruited and attended either in primary care health centers or on the premises of the research group at the University of the Balearic Islands. The call to enroll in the study was carried out through an oral announcement by the health personnel and/or by the researchers, as well as through the dissemination of announcements on posters and social networks. The total number of participants in the Ob-IB study was 490. All of them met the inclusion criteria (mainly, ≥18 years old who read and signed the informed consent) and did not report any of the exclusion criteria to participate in the project (lactating, pregnant, and/or carriers of contagious diseases; see [33] for details). In addition, subjects who reported taking supplements that may affect the nutritional status of VA were not included in the analysis. A complete data set (nutritional, genetic, anthropometric, and cardiometabolic risk) was obtained from 455 adults (165 men and 290 women) and included in the analysis (see Section 4.7. for how the missing data were managed). This early study allowed for the identification of nutrient–phenotype and gene–nutrient–phenotype interactions that would then be specifically studied in the following ex vivo approach. OptiDiet-15 (IB 2569/15 PI) is an ex vivo interventional approach aiming to characterize nutrient–phenotype–genotype interactions and explain from a cellular point of view the associations observed in the Ob-IB study (see above). OptiDiet-15 includes data from 81 individuals (40 women and 41 men) enrolled in the Ob-IB study, additionally providing a blood sample for the ex vivo interventional study and further characterization. To minimize any bias, care was taken to select balanced groups concerning the number of participants of each sex and similar potential/expected confounding factors. Estimation of the minimum sample size for experimental subgroups (e.g., after segregating by genotypes) was based on the number of subjects tested in similar ex vivo studies performed under controlled conditions [34,35,36]. 

### 4.2. Cardiometabolic Health Assessment

From anthropometric and clinical variables collected from Ob-IB volunteers, the number of cardiometabolic risk (CMR) factors was calculated. Specifically, one point was assigned for each of the following alterations suffered: a high percentage of body fat (BF%), visceral obesity (VO), hypertension (HT), dyslipidemia (DL), and type 2 diabetes (T2D). Individuals who counted ≥2 CMR were considered at “high cardiometabolic risk”, while those with one or no CMR were classified at “low risk”. In detail, body fat percentage (BF%) and VO were taken by a trained researcher during a face-to-face interview with the volunteer. BF% was obtained using a bio-impedance device (Omron BF306, Hoofddorp, The Netherlands). BF% values of ≥25 (in men) and ≥33 (in women) were considered high [37]. Waist and hip circumferences were measured using a non-elastic measuring tape, and the waist–hip ratio (WHR) was obtained using [waist circumference/hip circumference (cm)]. Waist circumference and WHR were used to discern the population with VO, in accordance with the Spanish Society for the Study of Obesity (SEEDO) [38] cut-offs. Thus, individuals with ≥94 cm (men) or ≥80 cm (women) of waist circumference plus WHR ≥ 0.90 (men) or ≥0.85 (women) were classified as having VO. Regarding clinical data, individuals were classified as HT, DL, and/or T2D if they reported being diagnosed with or taking medication for hypertension, dyslipidemia, and/or type 2 diabetes, respectively. 

### 4.3. Dietary Intake and Physical Activity Evaluation

The habitual intake profile of the volunteers’ diet was assessed by performing up to three 24 h recall reports. Then, the dietary software DIAL v2.0 (Alce Ingeniería, Madrid, Spain) was used to estimate the intake of total energy and nutrients, including VA, retinol, and carotene. The estimated VA intake is calculated by the DIAL with the following formula: [(µg of retinol) + (carotenoids with vitamin activity)/6] [39]. The estimation of the physical activity level was reported by the volunteers using the short version of the International Physical Activity Questionnaire (IPAQ) [40]. Data from the IPAQ is expressed as the metabolic equivalent of a task (MET/day), with one MET equivalent to 3.5 mL of consumed O_2_ per kg body weight per minute. 

### 4.4. Blood Sample Collection, Peripheral Blood Mononuclear Cell Isolation, and Ex Vivo Treatment

Peripheral blood mononuclear cells (PBMCs) extraction, isolation, and ex vivo treatment are described in more detail in previous work [36]. Briefly, around 20–30 mL of whole blood was collected in vacutainer-EDTA tubes from each OptiDiet-15 study volunteer after an overnight fasting period. Then, blood samples were diluted (1:1) with phosphate-buffered saline (PBS), and Ficoll-Paque Plus (GE Healthcare Bio Science, Barcelona, Spain) was carefully added on top of the mix. The blood cell density gradient was obtained by centrifugation for 30 min at 400× *g* at 20 °C (without brake). The specific gradient layer containing PBMCs was collected and washed twice with PBS. Afterward, ≥22 × 10^6^ cells were collected from the total amount and activated with CD3/CD28 magnetic beads (Life Technologies, Madrid, Spain). Then, cells from each subject were set in 22 wells of a 24-well plate, with 10^6^ cells per well, and maintained in suspension in culture medium RPMI-1640 (Sigma-Aldrich, St Louis, MO, USA), supplemented with 10% fetal bovine serum (FBS) (Gibco Biosciences, Dublin, Ireland), L-glutamine (1%) (Sigma-Aldrich, St Louis, MO, USA), and penicillin-streptomycin (100 units/mL and 100 µg/mL, respectively) for 48 h at 37 °C and 5% CO_2_. Ex vivo treatments consisted of incubating cells with all-trans RA (1.0 µM), all-trans R-OH (1.0 µM), or β-carotene (0.5 µM). The concentration of treatments was selected in terms of molar vitamin A value (considering that one mole of β-carotene is molecularly equivalent to two molecules of retinol [41]). Otherwise, control cells (C) were incubated with the vehicle used in treatments (DMSO). All treatments were acquired from Sigma (Sigma-Aldrich, St. Louis, MO, USA).

### 4.5. RNA Extraction and Gene Expression Selection and Analysis

PBMCs were isolated from the culture medium after 48 h of treatment. Total RNA extraction was performed using Direct-zol RNA Mini-Prep (Zymo Research Corp, Irvine, CA, USA). RNA was retro-transcribed (RT) to cDNA using a commercial kit (Bio-Rad Laboratories, Madrid, Spain) and a 2720 Thermal Cycler (Applied Biosystems, Madrid, Spain) following the manufacturer’s guidelines. RNA quantification was carried out with the spectrophotometer Nanodrop 1000 (Thermo-Fisher Scientific, Waltham, MA, USA). Gene expression was performed by real-time PCR (qPCR) using Power SYBR Green Master Mix (Applied Biosystems, Madrid, Spain), as described in more detail elsewhere [33]. Primers were designed by researchers involved in the OptiDiet-15 study and were purchased from Sigma Genosys (Sigma-Aldrich Química SA, Madrid, Spain). Gene expression analyses were based on the threshold cycles (Ct) of each determination using StepOne Software v2.0 (Applied Biosystems, Madrid, Spain), and data were normalized with the housekeeping gene Ribosomal Protein Large P0 (*RPLP0*). The mRNA levels of control and treated cells are expressed as their relative percentages with respect to the mean of the entire sample population (*n* = 81).

### 4.6. DNA Extraction and Genotype Analysis

The presence of rs5888 (*SCARB1*), rs1761667 (*CD36*), rs659366 (*UCP2*), rs9939609 (*FTO*), and rs1800629 (*TNFA*) was assessed in the subjects characterized. Genetic analysis was performed from saliva-isolated genomic DNA using specific SNP probes (purchased from Tib Molbiol, Berlin, Germany) following the conditions described in [13]. The genetic influence of SNP in the Ob-IB cohort was assessed, taking into account carotene tercile intake and CMR as response variables, using the online tool SNPStats [42]. To analyze haplotypes sufficiently represented in the studied population, separating the analysis for men and women, the top 10 most frequent genetic combinations were considered, and the most common haplotype in each sex was adopted as the reference. The mean differences of the CMR reference versus haplotype (and the 95% CI) were calculated in subjects allocated to the opposite extreme terciles (T1 and T3) of carotene intake. This allowed the identification of the haplotype(s) associated with a potentially worse response to low carotene intake. 

The interactions between the genetic load associated with higher CMR in cases of low intake of carotene in Ob_IB cohort and with gene expression determined in the OptiDiet-15 cohort were assessed. Men were classified by the number of T alleles for the *SCARB1* and *FTO* variants (which were associated with a higher CMR depending on the level of carotene intake). In the case of women, the number of T alleles carried for the *SCARB1* variant plus the A alleles of the *CD36* polymorphism was also taken into consideration. The cut-off was set at 3 alleles of risk. Consequently, subjects were divided into two genetic groups (low and high). Low genetic load: <3 alleles associated with a potentially better cardiometabolic response; high genetic load: ≥3 alleles associated with the adverse or non-responder phenotype. Then, the differential gene expression—in response to BC treatment in PBMCs—according to sex and genetic groups was studied. 

### 4.7. Statistical Analysis

Descriptive data from OptiDiet-15 and Ob-IB studies are shown as a specific mean (or percentage) ± standard deviation (SD) in tables and expressed as the mean ± standard error (SE) in graphs. The evaluation of the parametricity—normality and homogeneity of variance distribution—of variables was carried out by the Kolmogorov–Smirnov and Levene tests, respectively. Group comparisons and statistical assessments of associations were carried out with parametric statistical tests if the normal distribution of the variable was confirmed. Variables that did not meet parametric rules were logarithmically (log_10_) transformed. CMR values were used as a quantitative variable and to dichotomize the population according to whether they suffered two or more CMR factors simultaneously (high risk) versus low risk. Sex-specific terciles of the reported intake of VA, retinol, and carotenes were used to analyze associations with CMR factors as a quantitative variable by linear regression analyses adjusted by age, energy intake, and level of physical activity. Likewise, associations between these nutritional variables and CMR (high or low) were evaluated by logistic regressions adjusting for confounding variables (specifically indicated in each table/figure caption). Linear and logistic regression results were expressed as standardized regression coefficients (β) and odds ratios (OR), respectively. The measure of variation for both types of regressions is indicated by the confidence intervals (CI, lower–upper). All the above-mentioned statistical analyses were performed using SPSS v27.0 (IBM, Chicago, IL, USA), and statistical significance was set at *p* < 0.05. For the regression analyses, up to 10 volunteers (2.4% of men and 2.1% of women) presented missing data. Consequently, the subject with the specific variable missing was excluded from the test. For haplotype analyses, the log-additive model was assumed, the threshold for statistical significance was set at 0.05, and the model was adjusted for the covariates age, level of physical activity, and energy intake. Up to 12 individuals from the Ob-IB study presented missing data in any of the genotypes analyzed. These individuals (2.4% of men; 6.1% of women) were not included in the haplotype analyses.

The effect of the ex vivo treatments on PBMC gene expression was assessed by comparing the relative gene expression of treated cells with control cells (incubated with DMSO), and the statistical analysis was performed by a paired Student’s *t*-test. Missing or outsider data in the statistical analyses of gene expression led to the non-inclusion of the individual.

## 5. Conclusions

Cardiometabolic health is sustained by various factors, many of which are modulated by diet and lifestyle. In the present study, the combination of cross-sectional and ex vivo approaches showed differential cardiometabolic outputs related to VA precursors. Dietary source and adequacy, together with individuals’ genotype and sex, influence metabolic flexibility and may affect cardiometabolic health. Findings from this study highlight the need to tailor the intake of VA and precursors according to the particularities of each person to promote a healthier cardiometabolic state through a focused and integrated approach.

## Figures and Tables

**Table 1:** General characteristics of Ob-IB and OptiDiet-15 cohorts ^1^.

	Ob-IB Cohort			OptiDiet-15 Cohort		
	All	Men	Women	All	Men	Women
	Mean (SD)	Mean (SD)	Mean (SD)	Mean (SD)	Mean (SD)	Mean (SD)
	*n* = 455	*n* = 165	*n* = 290	*n* = 81	*n* = 41	*n* = 40
**General characteristics**						
Age (years)	36 (15)	37 (17)	35 (15)	36 (14)	36 (16)	36 (12)
Female (%)	63.7			49.4		
**Anthropometric features**						
BF%	29.5 (8.98)	24.5 (8.08)	32.4 (8.20)	30.6 (9.50)	25.1 (7.39)	36.3 (7.95)
Waist (cm)	83.8 (15.8)	92.2 (15.2)	79.1 (14.1)	86.3 (15.9)	89.0 (14.2)	83.5 (17.2)
WHR	0.87 (0.09)	0.93 (0.08)	0.84 (0.09)	0.87 (0.12)	0.92 (0.10)	0.83 (0.12)
**Prevalence of cardio-metabolic risk factors**						
High-BF (%)	47.0	46.1	47.6	51.9	41.5	62.5
VO (%)	34.1	35.8	33.1	40.7	39.0	42.5
HT (%)	9.40	14.2	6.67	12.3	17.1	7.50
DL (%)	11.2	11.7	10.9	6.17	12.2	0.00
T2D (%)	5.39	8.07	3.87	7.41	12.2	2.50
CMR (number)	1.07 (1.19)	1.17 (1.36)	1.01 (1.07)	1.19 (1.22)	1.22 (1.42)	1.15 (0.98)
≥2 CMR factors (%)	30.6	33.5	28.9	34.6	36.6	32.5
**Physical activity**						
METs/day	278 (260)	298 (287)	266 (244)	247 (192)	212 (161)	283 (216)
**Main nutritional characteristics**						
Energy intake (kcal/day)	2006 (459)	2227 (519)	1880 (366)	1959 (400)	2119 (378)	1796 (358)
Carbohydrate (%EC)	42.3 (10.0)	42.4 (10.1)	42.3 (10.0)	42.7 (9.17)	42.0 (7.70)	43.4 (10.5)
Fat (%EC)	37.7 (9.38)	37.3 (9.50)	38.0 (9.32)	37.3 (8.37)	37.4 (7.92)	37.3 (8.90)
Proteins (%EC)	16.0 (3.97)	16.0 (3.34)	16.1 (4.30)	15.9 (3.17)	15.7 (2.67)	16.1 (3.63)
Vitamin A (µg/day)	924 (2317)	1108 (3639)	819 (942)	564 (201)	569 (165)	558 (235)
Tercile 1	≤516	≤504	≤525	≤459	≤459	≤446
Tercile 3	>841	>842	>852	>647	>661	>649
Retinol (Eq/day)	474 (2285)	686 (3637)	353 (811)	250 (133)	253 (113)	248 (152)
Tercile 1	≤206	≤228	≤195	≤170	≤208	≤161
Tercile 3	>361	>368	>349	>310	>294	>324
Carotenes (µg/day)	2133 (1966)	1920 (1665)	2254 (2112)	1557 (1115)	1549 (1011)	1565 (1225)
Tercile 1	≤1013	≤870	≤1153	≤1013	≤1037	≤971
Tercile 3	>2295	>2146	>2414	>1757	>1705	>1859

^1^ Data are presented as means and standard deviations (SD) or as percentages (%). BF%, body fat percentage; WHR, waist–hip ratio; VO, visceral obesity; HT, hypertension; DL, dyslipidemia; T2D, type 2 diabetes); CMR, cardiometabolic risk; ≥2 CMR factors, population % with two or more metabolic alterations; METs, metabolic equivalents; EC, energy contribution; Eq, retinol equivalents.

**Table 2:** Haplotype analyses and associations with cardiometabolic risk depending on carotene intake ^1^.

Haplotype	Frequency	T1	(95% CI)	T3	(95% CI)
Men					
**C**AC**A**G	0.132	Ref	0.00	Ref	0.00
TGCTG	0.082	−0.06	(−0.26–0.13)	−1.05	(−1.19–−0.92)
CGCTG	0.077	0.06	(−0.08–0.20)	−1.02	(−1.13–−0.91)
TATTG	0.076	−1.30	(−1.49–−1.11)	−1.05	(−1.20–−0.90)
CGCAG	0.065	−0.59	(−0.69–−0.49)	−0.49	(−0.55–−0.42)
TGTTG	0.058	−0.43	(−0.48–−0.38)	−0.22	(−0.25–−0.18)
TACAG	0.058	−0.51	(−0.72–−0.30)	−0.68	(−0.81–−0.55)
CACTG	0.055	−1.48	(−1.58–−1.37)	−0.94	(−1.03–−0.86)
CATTG	0.051	0.15	(0.09–0.22)	−0.05	(−0.10–−0.00)
**T**AC**T**G	0.050	**0.53**	**(0.49–0.56)**	−0.16	(−0.18–−0.13)
Women					
CGCAG	0.103	Ref	0.00	Ref	0.00
CACTG	0.100	0.62	(0.42–0.82)	−0.98	(−1.12–−0.83)
TACTG	0.087	−0.25	(−0.46–−0.05)	−1.44	(−1.60–−1.29)
TGCTG	0.073	0.40	(0.19–0.61)	−0.56	(−0.72–−0.40)
CATTG	0.061	−0.01	(−0.24–0.22)	−0.98	(−1.14–−0.83)
TATTG	0.058	−0.09	(−0.32–0.15)	−0.76	(−0.93–−0.59)
TGCAG	0.056	0.20	(0.07–0.33)	−1.10	(−1.20–−1.00)
CGTTG	0.054	0.09	(−0.03–0.20)	−1.73	(−1.81–−1.66)
**TA**CAG	0.051	**0.92**	**(0.77–1.07)**	−0.35	(−0.44–−0.25)
CGCTG	0.051	−0.10	(−0.18–−0.03)	−0.68	(−0.74–−0.61)

^1^ List of common allele combinations (>4%) of rs5888 (C/T, on *SCARB1*), rs1761667 (A/G, on *CD36*), rs659366 (C/T, on *UCP2*), rs9939609 (A/T, on *FTO*), and rs1800629 (G/A, on *TNFA*) in men (top) and women (below). Combination frequency is expressed as parts per one. For each allele combination, the difference between CMR shown on the most common haplotype (ref) and observed for terciles (T1 and T3) of carotene intake is expressed as mean differences and 95% CI (lower–upper). The haplotypes, in men and women (separately), associated with the highest cardiometabolic risk at the lower tercile (T1) of carotene intake in comparison with the reference haplotype are indicated in red. Differential alleles between the reference haplotype and the ones associated with the highest cardiometabolic risk are highlighted in bold.

## Data Availability

The datasets generated during the current study are available from the corresponding author on reasonable request.
